# Carcass and meat traits of bubaline finished on sugarcane-based diets supplemented with spineless cactus as a replacement for wheat bran

**DOI:** 10.5713/ab.20.0825

**Published:** 2021-08-24

**Authors:** Christiano Raphael de Albuquerque Borges, Francisco Fernando Ramos de Carvalho, Maria Luciana Menezes Wanderley Neves, José Diógenes Pereira Neto, Guilherme Heliodoro Pedroso Vieira, Ricardo Alexandre Silva Pessoa

**Affiliations:** 1 Department of Animal Science, Federal University of Amazonas - UFAM, Parintins 69152-240, Brazil; 2 Department of Animal Science, Federal Rural University of Pernambuco - UFRPE, Recife 52171-900, Brazil

**Keywords:** Buffalo, Meat Tenderness, Nutrition, Tissue Composition

## Abstract

**Objective:**

An experiment was conducted to evaluate the effects of increasing levels of spineless cactus (0%, 33%, 66%, and 100%) used as a substitute for wheat bran in buffalo diets on quantitative and qualitative traits of the meat and carcass.

**Methods:**

Twenty Murrah buffaloes at 18 months of age, with a mean initial weight of 292.9±57.3 kg, were randomly allocated to four treatments with five replicates. The animals were slaughtered after 90 days in the feedlot. The effects of spineless cactus as a replacement for wheat bran in the diet of the buffaloes on the carcass and meat traits, slaughter weight, carcass yield and carcass measurements were studied.

**Results:**

Increased spineless cactus levels led to linear reduction in average daily gain, slaughter weight, hot and cold carcass weight, compactness index and in the amount of muscle in the carcass, and there is no difference between the control treatment and the 33% replacing level for these parameters. The quality of the meat was not influenced by the treatments.

**Conclusion:**

Spineless cactus can replace wheat bran by up to 33% in sugarcane-based diets for buffaloes, without influencing quantitative and qualitative traits of the meat and carcass.

## INTRODUCTION

Although there is not a specific demand for buffalo meat in Brazil, buffaloes are slaughtered and marketed daily as cattle due to the anatomical similarities between their carcasses. As such, the product reaches the consumer’s table as “beef”. This strategy allows for a rapid flow of bubaline meat in an already structured chain.

In the northeast region of Brazil, which holds the second largest herd in the country, buffalo farming is based on the use of natural grasslands. However, during the dry season, sugarcane tops (*Saccharum officinarum*, L) is the main roughage source to those animals due to its low cost and broad availability in the region.

The known limitations to the use of sugarcane (high levels of high non-rumen degradable fiber) may negatively interfere with dry matter (DM) intake, requiring the adoption of concentrate supplementation to ensure satisfactory performance. Nevertheless, concentrate feedstuffs can account for around 65% of animal feeding costs [1]. In this respect, spineless cactus (Nopalea cochenillifera Salm Dyck) may be to replace the energy concentrate, for its contents of non-fibrous carbohydrates (NFC) and total digestible nutrients (TDN).

Given the above-described scenario, the present study proposes to examine the effects of replacing the wheat bran of the diet by spineless cactus on performance and carcass and meat traits of buffaloes.

## MATERIALS AND METHODS

### Animal care

The experimental procedure was approved by the Institutional Animal Care and Use Committee at Rural Federal University of Pernambuco (065/2015).

### Location, animal, diets, and experimental treatments

The study was conducted in the facilities of the Federal Rural University of Pernambuco (UFRPE), in the state of Pernambuco, Brazil. Twenty dewormed uncastrated Murrah buffaloes at an average age of 18 months, with an average initial weight of 292.9±57.3 kg, were confined in individual stalls with automatic drinkers and a feeder.

All evaluations performed in the meat took place at the Meat Quality Laboratory at the Department of Animal Science of the same institution.

Both slaughter and carcass morphometric measurements were performed at a local meat-packing plant.

All experimental diets were formulated to allow for a daily weight gain of 800 g, based on the requirements estimated by Paul et al [2], Kearl [3] and Rangel et al [4].

The basis of the diet consisted of sugarcane cut at 487 days of age. After discarding the leaves, only the sugarcane top was used and processed in a forage machine, reducing particles to 1 cm. The spineless cactus was cut at 2 years of age. Daily it was crushed in a forage machine and incorporated into the other ingredients before feeding. Urea and ammonium sulfate were added to the diets to adjust the crude protein (CP) of the spineless cactus. Diets were offered as total mixed rations twice daily in two equal meals at 0800 h and 1600 h for *ad libitum* intake.

Dietary treatments consisted of a control diet with wheat bran and without spineless cactus, and four diets with spineless cactus replacing 33%, 66%, and 100% wheat bran, respectively (Table 1).

### Slaughter and samplings

Throughout the 90 days in the feedlot, the animals were weighed fortnightly, always after an 18-h feed-deprivation period. At the end of this period, the buffaloes were slaughtered according to the standard procedures of the municipal slaughterhouse in São Lourenço da Mata, PE, Brazil. Once weighed, the carcasses were sawn lengthwise. For uniformity, all assessments were performed on the left half of the carcass.

The morphometric measurements were performed according to the method described by Butterworth et al [5]. A section was made between the 8th and 13th ribs from which samples were collected, weighed, and cooled at 4°C for 24 h. Subsequently, the pH, temperature and weight of those samples were recorded. From those sections, another section was made, and samples were collected between the 9th and 11th ribs to estimate the physical and chemical composition of the carcass, following the method recommended by Hankins and Howe [6]. Between the 12th and 13th ribs, the *Longissimus dorsi* muscle area was outlined on tracing paper to measure loin-eye area by the grid method and measure backfat thickness (BFT).

The samples between the 12th and 13th ribs, corresponding to the *longissimus dorsi* muscle, were identified, weighed, and used for the physical analysis of the meat.

### Laboratory analysis

Dried feed, orts, and fecal samples were analyzed for DM, organic matter, ash, CP, ether extract (EE), neutral detergent fiber (NDF), and acid detergent fiber according to standard procedures AOAC [7]. Non-fibrous carbohydrates were calculated as follows according to Hall [8]: NFC (g/kg) = 1,000− ([CP − urea derived CP + urea]+NDF+EE+ash). Total carbohydrates (TC) were calculated according to the equation proposed by Sniffen et al [9], where: TC = 100−(% PB+ % EE+% ash). The TDN were obtained by TDN (%) = digestible non-fiber carbohydrate + digestible CP + (digestible EE×2.25) + digestible NDF [10]. TDNI (kg/d) = digestible CP intake + (digestible EE intake×2.25) + digestible aNDFap intake + digestible NFC.

The section between the 9th and 11th ribs was dissected, and the proportions of muscle, adipose, and bone tissue contained therein were determined. Subsequently, these tissues were ground and pre-dried at 55°C to 60°C for 72 h to obtain dry fatty matter (FDM). Subsequently, the FDM was pre-degreased through successive washes with petroleum ether to obtain the pre-fat dry matter (PDDM). Then, the samples were ground in a ball mill for further analysis and quantification of the contents of EE and nitrogen (N), according to AOAC procedures [7]. The protein content (P) was calculated by multiplying the nitrogen content by 6.25. The fat removed in the pre-degreasing was calculated as the difference between FDM and PDDM and added to the results obtained for the residual EE in PDDM to determine the total fat content. The physical and chemical compositions of the section between the 9th and 11th ribs were used to estimate the physical and chemical compositions of the carcass according to the Hankins and Howe [6].

Meat color was determined using a KONICA MINOLTA portable colorimeter (model CR400), according to Furtado et al [11]. A 2 g aliquot was collected from each *longissimus dorsi* sample to evaluate water-holding capacity (WHC), following the method proposed by Hamm [12]. Two 2.5 cm-thick steaks were extracted from the *longissimus dorsi* and used in the evaluations of cooking loss and shear force, following the method described by Wheeler et al [13].

### Experimental design and statistics

The experiment was laid out in a randomized-block design with blocks arranged according to the animals’ initial weight. Four treatments (replacement levels) were tested, with five replicates each.

The variables were analyzed based on the following statistical model:


$$Y_{ijk}=\mu +T_{i}+B_{j}+e_{ijk},$$

Where *Y_ijk_* is experimental response measured in treatment *i*, block *j* and replicate *k*; *μ* is overall constant; *T_i_* is effect of treatment *i* (*i* = 1, 2, 3, or 4); *B_j_* is effect of block *j*; and *e_ijk_* is random error associated with each observation, NID assumption (0; σ^2^).

The data were subjected to variance and regression analysis and the levels were decomposed orthogonally of the square sums associated with the variation in linear and quadratic. The means were compared by the Dunnett test with significance p<0.05. The MIXED procedure of SAS (Statistical Analysis System) software was used in all analysis.

## RESULTS

### Performance and carcass characteristics

The diets led to a linear decrease in average daily weight gain (p<0.0001), slaughter weight (SW) (p<0.0001) and hot (p = 0.0006) and cold (p = 0.0008) carcass weights, however, it did not affect the hot and cold carcass yields (p>0.05). Except for the carcass compactness index (p = 0.0018) and leg length (p = 0.0214), no morphometric measurements on the carcass were affected (p>0.05) by the replacement of wheat bran by cactus in the diets. These two parameters showed a decreasing linear effect, suggesting that the muscle deposition in the carcasses reduced with the replacement of wheat bran. Despite the linear reduction observed in these parameters, the control and 33% replacing level did not differ from each other (Table 2).

### Composition of the carcass

The amount of adipose tissue in the carcasses did not differ between treatments (p>0.05), however, lower muscle tissue amount was observed on 66% and 100% replacing levels and the diet with 33% replacing level did not differ from control (Table 3). The control treatment showed a greater amount of bone tissue in relation to the other treatments (p<0.05).

The diets did not influence the chemical composition of the carcass (% or kg), except for the content of water (p<0.001) and protein (p = 0.0027) that decreased with the replacement of wheat bran for spineless cactus (Table 3), these components differed from the control treatment in the levels of 66% and 100% of replacement (p<0.05).

### Physical characteristics of the meat

The WHC, cooking loss and shear force values were not affected (p>0.05) by the diets. As regards meat color, only the yellow color spectrum (b*) was influenced (p = 0.014) by replacement of wheat bran for spineless cactus (Table 4), with a quadratic response (Ŷ = 10.48137+0.06680×X−0.00058919×X^2^), with greater intensity (b* = 12.37) at the 56.69% level. The treatments did not promote significant differences in lightness and red color (p>0.05).

## DISCUSSION

### Performance and carcass characteristics

Considering that the diets were formulated to meet the daily gain needs of 0.800 kg, only the control diet and 33% replacement provided such gain (Table 2). In addition, it was observed that the control diet provided a total weight gain of 94.4 kg, which is more than twice as that achieved with the treatment in which wheat bran was fully replaced by cactus (46.4 kg; Table 2). A negative effect on the average daily gain and feed conversion of dairy heifers fed the same treatments as those tested in the current study was reported by Monteiro et al [14].

The observed differences in SW explain the decrease in carcass weight. When SW is standardized, significant differences are seldom observed in carcass weight [15]. The reduction in carcass weights did not reflect in hot or cold carcass yields. Changes in gastrointestinal tract size, fasting time and weight of non-carcass components can influence carcass yield [16], which might have caused the similarity between the observed values in this study. Cold carcass yields similar to those found in the present study were observed by Vaz et al [17]. However, higher yields were described in other studies [18,19]. The use of carcass yield and its precision as an indicator of performance or meat production may be questionable, since higher yields are almost always obtained with fatter animals with a smaller edible portion.

Although the BFT did not differ significantly among treatments, the values found were sufficient to avoid cooling loss and maintain the temperature after 24 h (Table 2). BFT is a trait of extremely important to prevent drying and shortening of muscle fibers (cold shortening) during cooling, acting as a thermal insulation material. This occurs in carcasses with BFTs lower than 3.0 mm, which compromise the quality of carcass and meat [1].

Considering that *Longissimus dorsi* area (LEA) is positively correlated with carcass weight, the decreasing LEA values may be attributed to the observed reduction in carcass weights. The LEA values obtained in this study ranged from 48.6 to 60.6 cm^2^. Loin-eye area ranges of 51.8 to 56.6 cm^2^ were reported in a study testing the inclusion of lipid sources in bubaline [20].

### Composition of the carcass

The differences detected for the weights of hot and cold carcasses (Table 2), where the heaviest carcasses were obtained in the control and 33% treatments, were due to greater muscle development and less bone retention (Table 3). These difference may explain the effects observed in the carcass compactness index (Table 2), which decreased with the replacement of wheat bran by palm and differed from the control from the substitution level of 66%. This index is strongly correlated with the amounts of muscle and adipose tissue deposited in the carcass [21]. The soft tissue/bone ratios obtained in the present study were 5.70, 6.18, 5.37, and 5.26 for the replacement levels of 0%, 33%, 66%, and 100%, whereas muscle/bone ratios were 4.10, 4.56, 3.91, and 3.72, respectively. If we associate those values with the fat/muscle tissue ratios (0.39, 0.35, 0.37, and 0.41) and consider only the carcass composition, we may state that the diets with up to 33% replacement provided more balanced carcasses, with most part of their edible portion composed of muscle rather than fat.

The ratios between bone, muscle, and fat change throughout the development of an animal simultaneously to the chemical components of its carcass (water, protein, fat, and minerals), both of which are influenced by factors like age, weight, breed, sex, and nutritional level [22].

As the muscle is composed of 75% water and approximately 19% to 25% protein, a linear reduction in the amount of these elements was expected since the muscle deposition also reduced linearly (Ŷ = 122.06502–0.22979×X). The mean carcass composition values estimated in the present study were considerably different from those found in the consulted literature [23], perhaps due to the different methodologies employed in evaluation.

### Physical characteristics of the meat

According to Maggioni et al [24], higher WHC values result in higher pH values in the meat, whereas more acidic meats show a lower WHC. In this study, the pH of the carcasses of all treatments was within the ideal range (5.4 to 5.8), which favored WHC. Meats with a low WHC usually exhibit greater losses during cooking, resulting dry and tasteless.

The average cooking loss among the treatments was 34.18% (Table 4), which is similar to the 34% and 32.7% reported by other researchers [16,23]. Shear force averaged 2.43 kgf, characterizing the meats from all treatments as tender, according to the standards presented by Shackelford et al [25], who proposed the following reference: shear force values above 9.0 kgf (tough meat), 6.0 to 9.0 kgf (medium tenderness) and below 6.0 kgf (tender meat).

At the time of purchase, color is the main factor considered by the consumer in choosing the meat, as it is normally related to product quality. The mean lightness (41.4) and red (20.0) values observed in this study indicate that all treatments provided red and bright meats (Table 4). This may be related to the age and the finishing system adopted in this experiment, since animals raised in feedlots and animals slaughtered at 18 to 24 months produce light and bright meat [26]. The level of substitution up to 56.69 intensified the yellow color of the meat. The quality of carotenoids present in the cactus likely contributed to the increased pigmentation of the marbled fat in the meat, since wheat bran is devoid of those pigmenting agents [27]. The yellow color of fat is not only related to an advanced age, but it can also be seen in young animals due to beta-carotenes present in the pasture and in some feedstuffs. In this regard, it is noteworthy that, in addition to pigmenting fat, beta-carotenes are beneficial to the human health [28]. The color parameters observed in the meats evaluated in this study are equivalent to those described in bovine meat by Muchenje et al [29]. This shows that the dark color attributed to bubaline meat is not characteristic of the species, but rather caused by factors such as higher myoglobin concentrations and a higher number of red muscle fibers, which result from the physical effort of grazing [30].

## CONCLUSION

We concluded that spineless cactus can replace wheat bran by up to 33% in sugarcane-based diets for buffaloes, without influencing quantitative and qualitative traits of the meat and carcass.

## Figures and Tables

**Table 1:** Proportion of ingredients and chemical composition of sugarcane-based diets supplemented with spineless cactus replacing wheat bran

Items	Replacement level (%)			

	0	33	66	100
Ingredient (g/kg as fed DM)				
Sugarcane	384.0	384.0	384.0	384.0
Soybean meal	40.0	40.0	40.0	40.0
Ground corn	245.0	245.0	245.0	245.0
Wheat bran	300.0	200.0	100.0	0.0
Spineless cactus	0.0	95.0	190.0	285.0
Urea+AS for cactus	0.0	5.00	10.0	15.0
Urea+AS for Sugarcane	16.0	16.0	16.0	16.0
Minerals	15.0	15.0	15.0	15.0
Chemical composition of diets (g/kg DM)				
DM	647.4	576.6	505.7	434.9
OM	937.1	936.3	935.6	934.8
Ash	41.99	58.03	74.07	90.10
CP	141.2	141.1	140.9	140.8
EE	39.3	36.3	33.3	30.3
NFC	430.1	459.7	489.2	518.8
NDFap	345.0	325.4	305.8	286.2
ADF	104.76	109.19	113.62	118.05
TC	692.31	697.24	702.16	707.09
TDN	640.0	650.0	670.0	700.0

DM, dry matter; AS, ammonium sulfate, g/kg as fed; OM, organic matter; CP, crude protein; EE, ether extract; NFC, non-fibrous carbohydrates; NDFap, neutral detergent fiber corrected for ash and protein; ADF, acid detergent fiber; TC, total carbohydrates; TDN, total digestible nutrients.

**Table 2:** Performance and carcass characteristics of buffaloes fed sugarcane-based diets supplemented with spineless cactus replacing wheat bran

Parameter	Replacement level (%)				SEM	Polynomial contrast	
	
	0	33	66	100		Linear	Quadratic
Initial body weight (kg)	307.2	289.5	296.3	292.9	-	-	-
Slaughter weight (kg)	401.60	376.68	350.76^*^	339.28^*^	21.7956	<0.0001	0.6831
Average daily gain (kg/d)	1.19	1.09	0.68^*^	0.58^*^	0.08577	<0.0001	0.9909
Feed conversion (kg)	7.58	8.88	14.16	12.88	1.8409	0.0218	0.4899
Hot carcass weight (kg)	201.20	187.40	173.81^*^	171.98^*^	12.7347	0.0006	0.4595
Cold carcass weight (kg)	195.33	182.06	167.64^*^	166.85^*^	12.5512	0.0008	0.4199
Cooling loss (%)	2.93	2.88	3.60	3.02	0.645	0.3487	0.4993
Hot carcass yield (%)	50.11	49.65	49.55	50.65	0.8476	0.6425	0.3013
Cold carcass yield (%)	48.64	48.23	47.77	49.13	0.9213	0.7703	0.2621
Carcass length (cm)	123.00	120.80	120.75	120.20	2.0383	0.2384	0.6891
Carcass compactness index (%)	1.59	1.50	1.39^*^	1.39^*^	0.05106	0.0018	0.3149
Leg length (cm)	76.00	76.40	75.25	73.00	0.9112	0.0214	0.1528
Leg thickness (cm)	25.40	24.20	23.75	23.20	1.097	0.02	0.521
Leg circumference (cm)	106.20	100.80	101.25	100.40	2.8581	0.0607	0.2651
Chest depth (cm)	43.00	41.80	42.67	41.80	1.8093	0.6003	0.7666
pH after 24 h	5.40	5.53	5.42	5.67	0.139	0.6791	0.2689
T 24 h (°C)	10.38	10.22	9.90	10.18	0.2881	0.4271	0.3989
*Longissimus dorsi area* (cm^2^)	60.60	54.80	50.75	48.60	4.432	0.0175	0.5888
Backfat thickness (mm)	8.84	6.65	7.65	9.13	1.1017	0.6956	0.1005

SEM, standard error of the mean.

* Differ from control by Dunnett test (p<0.05).

**Table 3:** Physical and chemical composition1) of the carcass of buffaloes fed sugarcane-based diets supplemented with spineless cactus replacing wheat bran

Parameter	Replacement level (%)				SEM	Polynomial contrast	
	
	0	33	66	100		Linear	Quadratic
Muscle tissue (%)	61.78	64.26	62.05	60.00	1.6272	0.2192	0.1061
Adipose tissue (%)	24.42	23.15	23.02	24.64	2.0016	0.9427	0.361
Bone tissue (%)	15.08	14.22	15.89	16.20	0.5596	0.0347	0.2406
Muscle tissue (kg)	120.99	117.58	103.94^*^	100.01^*^	7.562	0.0011	0.95
Adipose tissue (kg)	47.30	41.72	38.76	41.40	3.7572	0.1756	0.2246
Bone tissue (kg)	29.52	25.77^*^	26.56^*^	26.88^*^	1.4139	0.0329	0.0093
Water (%)	54.95	58.69	54.11	53.60	1.6821	0.1769	0.1408
Fat (%)	21.77	18.20	21.35	23.01	1.9277	0.2709	0.0719
Protein (%)	18.17	17.77	18.13	17.64	0.392	0.4721	0.8932
Ashes (%)	4.59	4.80	5.18	4.91	0.1465	0.0647	0.1207
Water (kg)	107.58	106.94	90.42^*^	89.44^*^	6.1852	<0.0001	0.9486
Fat (kg)	42.16	32.91	36.11	38.49	3.5722	0.5499	0.0613
Protein (kg)	35.55	32.50	30.35^*^	29.37^*^	1.9398	0.0027	0.4227
Ashes (kg)	9.03	8.76	8.70	8.16	0.61	0.1212	0.7049

1) Estimated by the equations of Hankins and Howe [6].

SEM standard error of the mean.

* Differ from control by Dunnett test (p<0.05).

**Table 4:** Physical traits of the meat from buffaloes fed sugarcane-based diets supplemented with spineless cactus replacing wheat bran

Parameter	Replacement level (%)				SEM	Polynomial contrast	
	
	0	33	66	100		Linear	Quadratic
Water-holding capacity (%)	63.03	64.73	59.07	60.98	1.7756	0.1028	0.9843
Cooking loss (%)	34.93	32.10	34.05	35.65	2.5491	0.5337	0.1929
Shear force (kgf/cm^2^)	2.55	2.06	2.54	2.57	0.244	0.6224	0.3203
Color							
L*	39.60	42.92	41.46	41.78	1.1739	0.2016	0.1042
a*	19.08	19.10	22.49	19.33	0.8638	0.3087	0.0977
b*	10.61	11.67	12.69^*^	11.15	0.4558	0.2092	0.014

SEM standard error of the mean.

* Differ from control by Dunnett test (p<0.05).
